# A Multistage Process Model of How a Person Who Currently Injects Drugs Comes to Assist Persons Who Do not Inject with Their First Injections

**DOI:** 10.3389/fsoc.2021.619560

**Published:** 2021-02-15

**Authors:** Don C. Des Jarlais, Kamyar Arasteh, David M. Barnes, Jonathan Feelemyer, Hayley Berg, Mait Raag, Ave Talu, Greete Org, Susan Tross, Anneli Uuskula

**Affiliations:** ^1^ Department of Epidemiology, College of Global Public Health, New York University, New York, NY, United States; ^2^ Department of Family Medicine and Public Health, University of Tartu, Tartu, Estonia; ^3^ Department of Psychiatry, Columbia University, New York, NY, United States

**Keywords:** persons who inject drugs (PWID), Estonia, Staten island, non injection drug use, New York City

## Abstract

Injecting drugs for the first time almost always requires assistance from an experienced person who injects drugs (PWID). While there has been moderate amount of research on PWID who assist with first injections, most of this research has focused on identifying characteristics of PWID who assist with first injections. We do not have a formal model that describes how the minority of PWID come to assist do so, while the majority never assist. Through comparison of persons who did or did not recently assist with first injections using data from PWID in Tallinn, Estonia (N = 286) and Staten Island, New York City (N = 101), we developed a formal multi-stage model of how PWID come to assist with first injections. The model had a primary pathway 1) of engaging in “injection promoting” behaviors, 2) being asked to assist, and 3) assisting. Statistical testing using odds ratios showed participation in each stage was strongly associated with participation in the next stage (all odds ratios >3.0) and the probabilities of assisting significantly increased with participation in the successive stages. We then used the model to compare engagement in the stages pre-vs. post participation in an intervention, and to compare persons who recently assisted to persons who had assisted in the past but had not recently assisted and to persons who had never assisted. Advantages of a formal model for how current PWID come to assist with first injections include: facilitating comparisons across different PWID populations and assessing strengths and limitations of interventions to reduce assisting with first injections.

## Introduction

The transition from non-injecting to injecting drug use greatly increases the likelihood of both individual and societal adverse consequences of illicit drug use. Compared to non-injecting use, injecting is more likely to lead to blood-borne virus transmission (HIV, hepatitis B and C), abscesses and other bacterial infections, fatal overdoses, and more rapid development of substance use disorders (Mathias, 1991; Griffiths et al., 1994; McBride et al., 2001; Mateu-Gelabert et al., 2005; Ochoa et al., 2005; Simmonds and Coomber, 2009).

The transmission of HIV through multi-person use (sharing) of needles and syringes for drug injecting deserves additional comment. During the 1980s, 1990s and 2000s HIV epidemics with seroprevalence reaching 30% or higher occurred in many parts of North America, Europe and Asia (Des Jarlais et al., 1994; van den Hoek et al., 1988; Wiessing et al., 2008). The development and implementation of “combined prevention and care for PWID” (primarily syringe service programs, opiate substitution treatment (OST) programs, and antiretroviral treatment as prevention) have led to “ending” many high prevalence HIV epidemics in North America and Europe (D.C. Des Jarlais et al., 2016). Despite these successes of combined prevention and care for PWID, multiple new outbreaks of HIV among PWID have occurred in the last decade. Outbreaks have occurred in Europe, Israel, Taiwan, and the US (Des Jarlais et al., 2020; Huang et al., 2019). Even with the highly effective tools of combined prevention and care, injecting drug use must be seen as a continuing threat for further transmission of HIV.

Injecting an illicit drug is a complicated and potentially dangerous procedure, and almost everyone who begins injecting requires the assistance of an experienced injector for a first injection (Rhodes et al., 2011; Kolla et al., 2015). We do have a relatively good understanding of the social-cognitive and interpersonal processes through which persons who use but do not inject drugs (non-PWID) are initiated into injecting (Rhodes et al., 2011; Kolla et al., 2015; Wenger, Lopez, Kral, and Bluthenthal, 2016; Guise, Horyniak, Melo, McNeil, and Werb, 2017). First, through their participation in the general illicit drug use subculture and their interactions with persons who inject drugs (PWID), non-PWID “normalize” injecting as a route of drug administration. Second, through further discussions with PWID and possible observations of PWID actually injecting, they become more interested in injecting, become motivated to try injecting, and then ask for assistance with their first injection.

We do not have a comparable process model for how some PWID come to provide assistance with first injections. Multiple cross-sectional quantitative studies of PWID who have provided assistance with first injections have found that only a minority, typically 10%–30% of PWID, have ever provided assistance with first injections (Crofts, 1996; Hunt et al., 1998; Bryant and Treloar, 2008; Rhodes et al., 2011; Bluthenthal et al., 2014; Rotondi et al., 2014). These quantitative studies have also identified a wide variety of factors that differentiated between PWID who assisted with first injections vs. PWID who did not assist with first injections, including: gender, age, race/ethnicity, educational attainment, frequency of heroin injection, and use of non-injectable drugs, (see Barnes et al., 2018) for a review.

Overall this is a long list of disparate factors. Some of the differences in the factors identified as distinguish between PWID who assisted vs. PWID who did not assist undoubtedly arise from methodological differences—different questionnaires, different time frames for having assisted—and some of the differences may arise from conducting the studies in different PWID populations. Nevertheless, given the common processes that underlie how non-PWID come to engage in first injections and the near universality of PWID receiving assistance with their first injections, one would expect that there may also be common process that lead current PWID to assist with first injections.

A formal conceptual description of how this minority of PWID come to provide assistance with first injections would permit statistical assessment of the fit of the model in different PWID populations and comparisons of the processes in the different populations. Formal specification of such a model should also provide insight into potential interventions to reduce the likelihood that current PWID would assist with first injections. Interventions that would reduce the likelihood of current PWID assisting with first injections could be extremely useful in reducing many of the adverse consequences of illicit drug use, including HIV and HCV transmission, overdoses, and bacterial infections (Werb et al., 2018).

We report here on the development of a multi-stage social process model for how some PWID came to recently assist with first injections while the great majority did not recently assist with first injections. We then apply the model to compare engagement in the different stages pre-vs. post participation in an intervention to reduce assisting with first injections, and then to differentiate between PWID who have never assisted with a first injection vs. those who did not recently assist but have assisted in the past. A final analysis identified characteristics of PWID who assisted with only one first injections and then did not assist with any other first injections.

The data used in developing the model come from the baseline (pre-intervention) data in a two-site clinical trial of an updated version of the “Break the Cycle” intervention (Des Jarlais, 2018; Des Jarlais et al., 2019).

## Methods

### Generating a Multi-Stage Process Model for Assisting with First Injections

Our model development was informed by review of the qualitative and quantitative literature on PWID assisting with first injections (Rhodes et al., 2011; Kolla et al., 2015; Wenger et al., 2016) and our previous research with persons who use drugs (both PWID and non-PWID). We also conducted qualitative research specifically to better understand why many PWID do not assist non-PWID with first injections (Barnes et al., 2018).

The led us to formulate requirements for a quantitative model that would describe how a few current PWID come to assist with a first injection and how the great majority of PWID do not assist with first injections:

1. The model would need to be consistent with the qualitative research on how PWID come to assist with first injections and with the qualitative and quantitative research on how non-injecting drug users come to inject for the first time.
2. Assisting with a first injection would not be a single, spontaneous event but rather the result of a multi-stage process of interactions between PWID and non-injecting drug users.
3. Engaging in each stage would be positively associated with engaging in the next stage and engaging in each successive stage would be associated with an increasing probability that a current PWID would assist with first injections.
4. Engaging in “injection promoting behaviors” (talking positively about injecting to non-injectors, injecting in front of non-injectors, and offering to assist with a first injection) would be a critical early stage in the process.
5. Assisting with a first injection is a consensual act, requiring explicit agreement between the PWID who provides assistance and the non-PWID who receives assistance.

We applied these requirements to the baseline data from participants in a two-site clinical trial of an updated version of the Break the Cycle intervention (Uusküla et al., 2018). Break the Cycle is an intervention based in social cognitive theory and motivational interviewing to reduce the likelihood that a current PWID will assist a non-PWID with a first injection. It was originally developed by Hunt et al., 1998 and later adapted by Strike et al., 2014 to use peers as the interventionists. The Hunt intervention consisted of questions in five different sections: the participant’s own initiation, their initiation of others, the risk from initiation for themselves and the initiate, identification of aspects of their own behavior that may inadvertently promote injecting, and generation and rehearsal of responses to a series of vignettes describing common initiation scenarios. Strike extended the intervention to include information on safe injection education and sources of syringes and injection equipment in the community, which was developed from the Canadian AIDS Treatment Information Exchange (Canadian AIDS Treatment Information Exchange, 2008).

### Clinical Trial Study

The full results of this clinical trial have been reported elsewhere (NCT 03502525) (Des Jarlais, 2018; Des Jarlais et al., 2019) so that only a brief description will be presented here.

### Participant Eligibility

PWID were eligible for the study if they were 18 or older, spoke Estonian or Russian (Tallinn) or English (Staten Island), reported having injected in the previous two months, and were able and willing to provide informed consent.

### Recruitment

#### Tallinn

Respondent driven sampling (RDS) (Heckathorn, 1997; Heckathorn, 2002) was used. The syringe exchange program Convictus served as the research site. After study participation, subjects were provided coupons for recruiting up to three peers to participate in the study.

#### New York City

Program staff on the Community Health Action of Staten Island (CHASI) mobile syringe exchange bus were made familiar with the eligibility criteria and referred potentially eligible participants to research staff on the unit based on a convenience sampling approach. Research staff screened the referrals.

### Study Procedures

After eligibility determination and informed consent, participants completed a face-to-face interviewer-administered structured questionnaire which lasted approximately 30 min. Questions elicited information on demographics, experiences with injection and other drug use, sexual risk behavior and use of various HIV/harm reduction-related services. The behavioral questions used a “in the past 6 months” time framework.

### Intervention

Immediately after the baseline interview, the PWID participated in a “Break the Cycle” intervention conducted by the interviewer, who had been trained in the intervention (Des Jarlais et al., 2019). The intervention session took 30–40 min. The intervention was aimed at enhancing current injectors’ motivation and skills to avoid helping non-injecting drug users transition to injection drug use. It was informed by two main approaches to behavior change: Social Cognitive Theory, which explains behavior change as the result of peer modeling, expectancies about the target behavior, and perceived self-efficacy to carry out the target behavior (Bandura, 1993); and Motivational Interviewing (MI) (Miller and Rollnick, 2012). MI is a client-centered approach that proceeds from the premise that almost all individuals have ambivalence about behavior change. MI is aimed at articulating and resolving that ambivalence in the direction of healthier behavior and pinpointing next action steps.

The intervention had seven main parts: 1) discussion of own first time injecting drugs; 2) discussion of injection “promoting” and “assisting” behaviors, and experiences with and attitudes toward these behaviors; 3) discussion of the health, legal, social, and emotional risks of injection (including a module on safe injection practices); 4) role-plays of behaviors and scripts for avoiding or refusing requests to help non-PWID inject for the first time; 5) role-plays of talking with other PWID about not encouraging non-PWID to start injecting; 6) discussion of coaching non-PWID in safer injection practices, should they feel helping is their best option; and 7) discussion of how naloxone can be used to reverse overdose.

### Measuring “Injection Promoting” and “Assisting with a First Injection” Behaviors

We developed and pre-tested specific question about attitudes and behaviors related to assisting with first injections. These questions included:

1. Engaging in “injection promoting” behaviors, defined as: 1) speaking positively about injecting to non-PWID, 2) injecting in front of non-PWID, and 3) offering to give a first injection. Separate questions were asked about each of these distinct behaviors.
2. Whether the participant had “assisted with a first injection,” defined as “explaining, or describing or demonstrating how to inject to a person who then injected for their first time,” or “injecting a person who had not injected before.” This was asked as a single question as our pre-testing indicated that many of these behaviors were performed within a single episode of assisting.

We asked questions on assisting both at the baseline interview and at the follow-up interview, which occurred approximately six months after the initial interview was conducted. Follow-up interview questions queried specifically on behaviors in the last six months (i.e. the period between the baseline interview/intervention and the follow-up interview only).

### RDS Weighting

For Tallinn, there were small difference between the RDS weighted and unweighted values (<3% for all major variables). We therefore used the unweighted data to facilitate comparisons with Staten Island.

### Missing and Inconsistent Data

Thirteen subjects from Tallinn and 2 subjects from Staten Island with missing or inconsistent data on injection promoting, being asked to assist, and assisting with first injections were excluded from the analyses.

### Honoraria

Participants were paid modest honoraria for their time and effort in the study, and in Tallinn, for recruiting additional participants.

### Audiotaping of Intervention Sessions

In order to monitor fidelity of the interventions and to obtain greater insight into how participants experienced the intervention, we audiotaped the intervention sessions. This was done with explicit approval of the participants, and they were cautioned not to use the names of any other persons they mentioned during the intervention sessions.

### Ethical Approval

Ethical approval for the study was obtained from the Ethics Review Board of the University of Tartu, Estonia and from Mount Sinai Beth Israel Medical Center and New York University School of Medicine Institutional Review Board in New York, United States.

## Results

### Demographics, Drug Use, and Factors Associated with Injection Promoting Behaviors

Table 1 presents demographic characteristics, drug use, and injection initiation related behaviors for the pre-intervention interviews of the participants used in developing the multi-stage model. The drug use related behaviors referred to the 6-month period prior to the interview. A total of 286 PWID were included from Tallinn and 101 were included from Staten Island.

**TABLE 1:** Demographics, drug use characteristics, and promoting behaviors among PWID in Tallinn and Staten Island, New York City.

	Tallinn		New York city	
	N	%	N	%
Total	286	100	101	100
Avg. age (SD)	33 (7)	—	44 (11)	—
Avg. years injecting (SD)	14 (6)	—	17 (14)	—
Gender				
Male	221	77	63	62
Female	65	23	38	38
Race or ethnicity				
Russian	230	80	—	—
Estonian	39	14	—	—
White	—	—	51	51
Black	—	—	22	22
Latinx	—	—	13	13
Other	17	6	15	15
Non-injecting drug use				
Any non-injected drug use	193	67	94	93
Speedball sniff/snort/smoked	—	—	46	46
Heroin sniff/snort/smoked	—	—	57	56
Fentanyl sniff/snort/smoked	99	35	4	4
Opiate analgesic pills swallowed	44	15	53	52
Cocaine sniff/snorted	—	—	41	41
Crack smoked	—	—	71	70
Amphetamines	43	15	19	19
Street methadone	28	10	33	33
Injecting drug use				
Heroin injected	—	—	96	95
Speedball injected	—	—	38	38
Cocaine injected	1	1	36	36
Fentanyl injected	205	72	4	4
Opiate analgesics injected	3	1	14	14
Amphetamines injected	185	65	—	—
Receptive sharing	40	14	9	9
Distributive sharing	67	23	9	9
Sexually active	242	85	79	79
Unsafe sex	178	74	25	25
Friends assisted w/1st injection	84	29	53	53
Likely to assist w/1st injection	67	36	14	14
Any promoting behavior^a^	81	28	38	38
Talked positively about injecting	20	7	25	25
Modeled injecting	74	26	25	25
Offered to inject	3	1	5	5
Helped inject last 6 months	12	4	12	12

^a^ Talking, modeling, offering to inject.

There are major differences between the Staten Island and the Tallinn subjects in almost all of the injecting and non-injecting drug use variables, to where standard statistical testing is not meaningful. The two samples clearly come from different drug using populations. Majorities of participants in both sites used non-injected drugs and thus were likely to have opportunities interact with non-PWID.

### Engaging in “Injection Promoting Behaviors”

Substantial percentages of the participants reported engaging in at least one “injection promoting” behavior in both sites—28% (81/286) in Tallinn and 38% (40/101) in Staten Island. We tested all factors (except assisting with a first injection) in Table 1 for associations with engaging in any promoting behavior. Table 2 shows factors that were significant in either or both of the two samples. It should be noted that “any non-injecting drug use” was strongly associated with engaging in promoting behavior for the Tallinn sample. “Any non-injecting drug use” was not statistically associated with promoting behavior in the Staten Island sample because almost all (94%) of the Staten Island participants reported non-injecting drug use. Thus, non-injecting drug use among the Staten Island participants did not distinguish engaging from not engaging in promoting behavior but should not be ruled out from being involved in promoting behavior. We considered these as “factors associated” with promoting injection but note that many of them were likely to be present before a PWID engaged in promoting behaviors, and thus may have served as causes for engaging in promoting.

**TABLE 2:** Factors significantly associated with injection promoting behavior^a^ among PWID in Tallinn and Staten Island, New York City.

Site	Staten Island, New York city			Tallinn Estonia		
Variable	OR	95% CI		OR	95% CI	
Age (continuous)				0.94	0.90	0.98
Gender						
Male (female: ref)	3.41	1.35	8.58			
Race/ethnicity						
Black (white: ref)	0.29	0.09	0.99			
Non-injection drug use						
Any non-injection drug use				2.61	1.44	4.97
Street methadone use	3.50	1.47	8.36			
Injection drug use						
Less frequent drug injection				1.79	1.01	3.13
Larger injection network size^b^	1.03	1.00	1.05	1.02	1.01	1.03
Receptive sharing	16.53	1.98	138.30	2.84	1.43	5.65
Distributive sharing	6.89	1.35	35.15	3.48	1.97	6.21
Friends who assisted w/1st injection	2.89	1.24	6.74	5.11	2.60	10.34
Endorsing likely to assist with first injection in future	5.27	1.52	18.27	2.85	1.65	4.94

^a^ Promoting behavior–talking positively about, demonstrating, offering to help with injecting.

^b^ Injection network size was categorized as “larger injection network size” when network size was greater than the median (7).

There were both similarities and differences in the “factors associated with injection promoting” across the two sites. The differences may reflect how variations in the local drug use culture feed into a common predominant pathway to assisting with first injections.

Across the two sites, the best predictor of which PWID engaged in promoting behavior was whether they exhibited 4 or more of these factors. In New York, 29/55 (53%) of the PWID who endorsed 4 or more factors promoted vs. 9/46 (20%) of the PWID who endorsed less than 4 factors (chi square = 11.7, *p* = 0.001). In Tallinn, 23/37 (63%) of PWID who endorsed 4 or more factors promoted vs. 53/236 (23%) of PWID who endorsed less than 4 factors (chi square = 24.3, *p* < 0.001). The odds ratios between having 4+ “associated factors” and engaging in injection promoting were in Tallinn (OR = 7.3, 95% CI 3.3–16.4) and Staten Island (OR = 4.6, 95% CI 1.9–11.3).

### Being Asked to Assist with a First Injection

Engaging in injection promoting behavior was strongly associated with being asked by non-PWID to assist with a first injection during the 6 months prior to the interview. In Staten Island, 24/38 (63%) who had promoted were asked by a non-PWID to assist with a first injection vs. 21/63 (33%) who had not promoted. In Tallinn, 31/86 (36%) of the PWID who had promoted were asked to assist vs. 28/202 (14%) who had not promoted. Both of these relationships between engaging in injection promoting and being asked to assist were substantial and statistically significant in Tallinn (OR = 3.5, 95% CI 1.8–6.6) and Staten Island (OR = 3.4, 95% CI 1.5–8.0).

### Assisting with a First Injection

Being asked to assist was strongly associated with actually assisting with a first injection; 21% (12/58) participants in Tallinn who were asked to assist assisted with a first injection and 27% (12/45) in Staten Island who were asked assisted. In neither site were there any participants who assisted who had not been asked to assist, so that ORs could not be calculated for assisting with being asked vs. assisting without being asked.

Whether participants who were asked to assist had engaged in injection promoting behavior in the 6 months prior to the interview was strongly associated with whether they assisted. In Tallinn, 10/30 of those who promoted and were asked did assist vs. 2/28 of those who did not promote and were asked (OR = 6.5, 95% CI 1.2–65.6). In Staten Island, 10/24 of those who promoted and were asked assisted vs. 2/21 of those who did not promote and were asked (OR = 6.8, 95% CI 1.1–70.3).

### Flow Diagrams and Probabilities of Assisting with a First Injection

**FIGURE 1:**
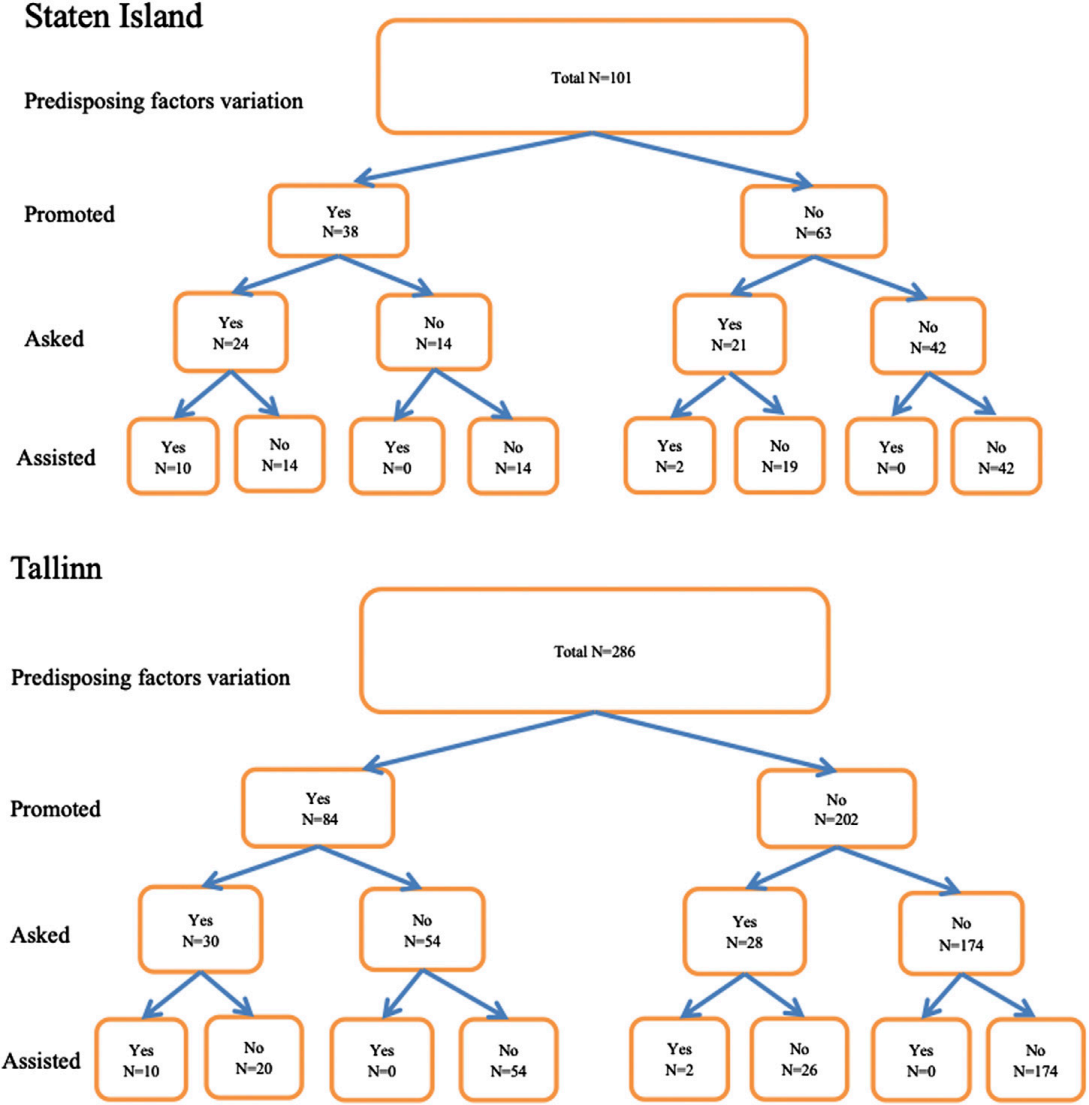
Flow diagrams of the different stages leading from engaging in injection promoting behaviors to actually assisting with a first injection at each site.

Figure 1 shows flow diagrams of the different stages leading from engaging in injection promoting behaviors to actually assisting with a first injection at each site. In both sites there was a predominant pathway (engaging in injection promoting behavior and then being asked to assist with first injection, noted in red) and a secondary pathway (not engaging in promoting behavior but being asked to assist, noted in black) leading up to actually assisting with a first injection.

As specified in the development of the model, engaging in additional stages (injection promoting behavior, being asked to assist) was associated with increases in the probability of assisting with first injections.

### For Tallinn

1. Participants who engaged in neither promoting nor being asked had a 0 (0/174) probability of assisting.
2. Participants who engaged in promoting had a 0.12 (10/84) probability of assisting.
3. Participants who both engaged in injection promoting and were asked to assist had a 0.33 (10/30) probability of assisting.

### For Staten Island

1. Participants who engaged in neither promoting nor being asked had a 0 (0/42) probability of assisting.
2. Participants who engaged in promoting had a 0.26 (10/38) probability of assisting,
3. Participants who both engaged in injection promoting and were asked to assist had a 0.42 (10/24) probability of assisting.

For both sites, the probability of assisting greatly increased with participation in the two stages of engaging in injection promoting and being asked to assist. Fisher exact test comparisons of the probabilities of assisting given neither promoting nor being asked vs. both promoting and being asked were statistically significant, *p* < 0.0001 for both Tallinn and Staten Island.

### Results II: Application of the Model to a Break the Cycle Intervention

Table 3 shows the pre-post intervention changes in the percentage of trial participants in the three stages of our multi-stage model. There was a statistically significant effect in reducing the percentage of participants who engaged in injection promoting behavior in Tallinn, but the reduction was not significant in Staten Island. As noted in Table 1, a very high percentage (93%) of the Staten Island participants reported non-injecting use of heroin, cocaine and prescription opioids, and thus were likely to have had many opportunities to engage in injection promoting behavior with non-PWID.

**TABLE 3:** Changes in outcomes for targeted behaviors.

	Tallinn	Tallinn^a^	Staten island	Staten island^a^
	(Pre)	(Post)	(Pre)	(Post)
	N = 230	N = 230	N = 64	N = 64
Any promoting behavior	33%	20%*	33%	28%
Asked to assist with first injection	18%	15%	44%	45%
Assisted with first injection	5%	1%*	15%	6%*

*p < 0.05.

^a^ Post measurement took place six months after baseline interview and intervention.

There was no reduction in being asked to assist with first injections in either site. Asking to assist with first injections is largely under the control of non-PWID, so it is probably unrealistic to expect that Break the Cycle interventions would have a significant effect on being asked to assist. Different interventions that focus on non-PWID are needed to reduce asking for assistance with first injections.

There were statistically significant reductions in both sites in the primary outcome of actually assisting with first injections. We attribute this effect to the intervention focusing existing motivations to not assist and to the role play practice of declining to assist when asked to assist.

Tables 4, 5 gives a comparison of endorsing facilitating factors for injection promoting, having engaged in promoting behaviors, and having been asked to assist among those who had never helped someone inject, those who had helped prior to previous 6 months, and those in the last 6 months among PWID in Staten Island. There was a consistent pattern with the never assisters being lowest, the previous but not recent assisters being intermediate, and the recent assisters being highest on all of these measures. Substantial numbers of the never assisters, however, did engage in injection promoting behavior and had been recently asked to provide assistance with a first injection.

**TABLE 4:** Comparison of promoting behaviors and being asked to help among those who had never helped someone inject, those who had helped prior to previous 6 months, and those who in the last 6 months among PWID in Staten Island.

	Never assisted	Assisted with first injection	
	N = 71	Assisted >6 months ago, N = 18	Assisted in last 6 months, N = 12
Mean of facilitating factors	3.6	4.2	5.3
Median of facilitating factors	3	4	6
	(N, %)*	(N, %)*	(N, %)*
Promoted	19 (26%)	9 (50%)	10 (83%)
Were asked for assistance	25 (35%)	8 (44%)	12 (100%)

**TABLE 5:** Comparison of promoting behaviors and being asked to help among those who had never helped someone inject, those who had helped prior to previous 6 months, and those who in the last 6 months among PWID in Tallinn.

	Never assisted	Assisted with first injection	
	N = 246	Assisted >6 months ago, N = 40	Assisted in last 6 months, N = 14
Mean of facilitating factors	3.3	4.3	4.6
Median of facilitating factors	3	4	5
Number who promoted (%)	61 (26%)	915 (38%)	11 (79%)
Number who were asked for assistance (%)	41 (15%)	6 (15%)	12 (86%)

## Discussion

The postulates and stages in our multi-step process model were derived partly from the literature, and thus are not unique to this analysis. We do believe, however, that the formal statement of the model has major advantages. A formal statement permits statistical examination of the associations between participating in the successive stages and in changes in the stage-associated probabilities of assisting with first injections. The model is thus “falsifiable.” If the associations between participating in successive stages in the predominant pathway had not been statistically significant, or if the probabilities of assisting with first injection had not increased with passage through the successive stages, we would have concluded that the model did not fit the quantitative data.

The formal statement of the model and the statistical analyses then permit a close comparison across sites. The probability analyses did show very strong similarities across the Tallinn and Staten Island PWID populations. These populations clearly varied in terms of drugs injected, race/ethnicity, extent of non-injecting drug use among PWID, and the pre-intervention rates of injection promoting behavior and assisting with first injections. The similarities in the fit of the data to the model in the two sites suggest that the model may be applicable to a wide variety of PWID populations. The strong similarities across the two sites in the numbers of factors associated injection promoting across PWID who never assisted, who assisted previously but not recently, and who assisted recently suggest similarities in time (since assisting) as well as similarities across the geographic sites.

As noted in the introduction, previous cross-sectional quantitative studies of characteristics of PWID who assist with first injections noted a variety of factors, including gender, age, race/ethnicity, educational attainment, frequency of heroin injection, and use of non-injectable drugs, without great consistency among the studies. Our model is consistent with these previous studies in terms of many of the factors associated with assisting. Our model differs from the multivariable models in the previous studies in that multivariable regression compares the strength of individual factors associated with assisting, and backward elimination will remove many correlated factors from the final model. Our model, in contrast, includes multiple stages so that an individual factor, e.g., non-injecting drug use, may be associated with progression to a later stage, e.g., injection promoting behavior. Our model includes the potential for different factors operating in different temporal stages of the process.

Finally, the formal statement of the model can also be used to assess the strengths and weaknesses of interventions to reduce the likelihood of a current PWID providing assistance with first injections. For these implementations of Break the Cycle in Staten Island and Tallinn, there were significant declines in declining to assist when asked to assist, and a significant decline in injection promotion in Tallinn. These changes are consistent with the motivational interviewing basis of the intervention to focus on and strengthen the participants’ existing motivation not to initiate others into injecting drug use.

The model also clarifies some weaknesses in the intervention. Promoting behavior was quite common among the Staten Island participants prior to the intervention and was not significantly reduced, and even though promoting behavior was reduced in Tallinn, it was still common post-intervention among the Tallinn participants. (20% reported engaging in injection promotion during follow-up.)

The lack of any reduction in being asked for assistance indicates two other limitations of this version of Break the Cycle. First, it is likely that the intervention would need to be strengthened and implemented on a very large scale to reduce injection promoting and the demand for assistance within a drug using population. Second, the repeatedly being asked to assist with first injections is likely to wear down resistance to assisting among some intervention participants PWID who would prefer to not provide assistance. Like many behavioral interventions, the effects of Break the Cycle may diminish over time. This could require either providing booster sessions for participants or implementing Break the Cycle on a sufficiently large scale within the PWID culture so that PWID would enforce norms against providing assistance with first injections.

### Potential Generalizations and Harm Reduction

We need to be extremely cautious in generalizing from just two sites but want to offer possible generalizations for future research on PWID who do assist with first injections. First, these PWID appear to be greatly involved in both injecting and non-injecting drug subcultures. They not only used non-injected drugs but also have large injecting networks. Second, they reported risky drug use. In both sites, receptive and distributive syringe sharing were associated with engaging in injection promotion. Assisting with a first injection may in itself be considered a health risk behavior. There are the immediate possibilities of a botched injection leading to a skin infection, of an overdose, and of HIV or HCV transmission if sufficient numbers of sterile syringes are not available. And, of course, there are the possibilities of multiple adverse health consequences if the initiate adopts injecting as a regular route of administration.

Given these multiple risks, PWID who assist with first injections would be a particularly appropriate group for engaging in harm reduction activities.

### Limitations of the Model

Several limitations of the present model should be noted. First, while the PWID populations in Tallinn and Staten Island are clearly different, these are only two sites. Some modifications of the model may be needed to describe how PWID come to assist with first injections in the very wide variety of PWID populations throughout the world. We suspect there may be possible local site differences in factors associated with engaging in injection promoting and possible additional secondary pathways to assisting.

Second, the model is currently based on cross-sectional data from PWID only. Incorporation of longitudinal dyadic data—from both the non-PWID being assisted with a first injection and from the PWID providing assistance—should extend and strengthen the model.

### Next Steps

Initiation into injecting drug use continues as a world-wide public health problem. The current “opioid epidemic” in the US (Scholl et al., 2019) is only the most recent example of rapid expansion of injection drug use. The multi-stage model described here and the clinical trial results of the Break the Cycle—Avant Garde suggest that there is very much that could be done to reduce initiation into injecting drug use. We would suggest the following as next steps:

1. Assessing fit of the model to data from additional PWID populations. If the model is found to apply to initiation into injecting drug use in a wide variety of situations, use the model to guide further research into reducing initiation.
2. Expansion and adaption of Break the Cycle type interventions to many additional areas.
3. Determination if reduced versions of Break the Cycle type intervention might still be effective so that the intervention might be easily implemented on a larger scale.
4. Assess sustainability of effects for Break the Cycle type interventions.
5. Integrate Break the Cycle interventions with interventions to increase NIDUs resistance to injection promoting behaviors. These should include greater access to substance use treatment (including methadone and buprenorphine) so that NIDUs do not initiate injecting because of financial pressures.
6. Socio-behavioral interventions to increase NIDU’s motivations to avoid injecting, such as the “Sniffer Project” (Casriel et al., 1990; ; Des Jarlais et al., 1992) also need to be further researched and then implemented on a public health scale.
7. With the COVID-19 epidemic, many health services for people who use drugs have moved to telehealth platforms. It would be important to determine if behavioral interventions, such as Break the Cycle, that utilized motivational interviewing can also be provided effectively through telehealth.

We believe that the multi-stage model developed here can be utilized to adapt interventions to different drug use settings and to assess the strengths and weaknesses of future interventions to reduce the likelihood that PWID will assist with first injections.

## Conclusion

We developed a formal multi-stage model of how a current PWID comes to provide assistance with first injections by non-PWID—through engaging in injection promoting behavior, being asked for assistance, and then providing assistance. The model can be subjected to statistical analyses and thus is “falsifiable.” The model fit quite well with data from two very different PWID populations, revealed strong similarities in the process of coming to assist with first injections in the two different sites, and can be used to assess strengths and limitations of interventions to reduce the likelihood that current PWID will provide assistance with first injections.

## Data Availability

**Restrictions apply to the datasets:** The datasets presented in this article are not readily available because there are participant identifiers contained within the dataset on a cohort of persons who use drugs. Requests to access the datasets should be directed to the primary author, Don Des Jarlais.
